# Exploring behavioral adjustments of proportion congruency manipulations in an Eriksen flanker task with visual and auditory distractor modalities

**DOI:** 10.3758/s13421-023-01447-x

**Published:** 2023-08-07

**Authors:** Linda C. Bräutigam, Hartmut Leuthold, Ian G. Mackenzie, Victor Mittelstädt

**Affiliations:** Department of Psychology, University of Tübingen, Schleichstrasse 4, 72076 Tübingen, Germany

**Keywords:** Conflict tasks, Eriksen flanker, Cross modal, Multimodal, Congruency effects, Delta plots, Cognitive control

## Abstract

The present study investigated global behavioral adaptation effects to conflict arising from different distractor modalities. Three experiments were conducted using an Eriksen flanker paradigm with constant visual targets, but randomly varying auditory or visual distractors. In Experiment 1, the proportion of congruent to incongruent trials was varied for both distractor modalities, whereas in Experiments 2A and 2B, this proportion congruency (PC) manipulation was applied to trials with one distractor modality (inducer) to test potential behavioral transfer effects to trials with the other distractor modality (diagnostic). In all experiments, mean proportion congruency effects (PCEs) were present in trials with a PC manipulation, but there was no evidence of transfer to diagnostic trials in Experiments 2A and 2B. Distributional analyses (delta plots) provided further evidence for distractor modality-specific global behavioral adaptations by showing differences in the slope of delta plots with visual but not auditory distractors when increasing the ratio of congruent trials. Thus, it is suggested that distractor modalities constrain global behavioral adaptation effects due to the learning of modality-specific memory traces (e.g., distractor–target associations) and/or the modality-specific cognitive control processes (e.g., suppression of modality-specific distractor-based activation). Moreover, additional analyses revealed partial transfer of the congruency sequence effect across trials with different distractor modalities suggesting that distractor modality may differentially affect local and global behavioral adaptations.

We are continuously confronted with an extensive amount of multimodal information (e.g., auditory and visual stimuli), with only a subset (i.e., targets) being relevant for our current task goal. Unfortunately, the capacity of the human information processing system is limited in a way that not all sensory information can be equally processed (Cowan, 1988). Furthermore, task-irrelevant information (i.e., distractors) can also harmfully interfere with target processing. To support flexible-goal-directed behavior, several possibilities have been suggested, like focusing attention on targets (e.g., Egner & Hirsch, 2005; Fischer et al., 2008; Lavie, 2010; Lavie et al., 2004; Notebaert & Verguts, 2008), suppressing distractors (e.g., Amer et al., 2016; Hasher et al., 1999; Stürmer et al., 2002; Treccani et al., 2018; Wühr & Ansorge, 2005), and adjusting processing based on learned environmental regularities (e.g., Braem et al., 2014, 2019; Chen et al., 2021; Logan & Zbrodoff, 1979; Schmidt, 2013a, b, 2019). However, the interplay of target and distractor processing across different modalities remains still relatively unexplored. In the present study, we hope to provide some further insights into this issue by exploring interference (e.g., response conflict) arising from different distracting modalities. Specifically, we examined global^1^ behavioral adjustments (i.e., proportion congruency effect) in the Eriksen flanker task under the presence of visual targets with either visual or auditory distracting information.

## The Eriksen flanker task

Conflict tasks are commonly used in the laboratory to shed light on the mechanisms underlying goal-directed behavior by presenting participants with task-relevant and task-irrelevant, potentially conflicting sources of information (Eriksen & Eriksen, 1974; Simon, 1990; Stroop, 1935). One of the most well-known conflict tasks is the Eriksen flanker task (Eriksen & Eriksen, 1974). In the most typical version of this task, participants are asked to respond to a central visual target while ignoring the surrounding irrelevant visual flankers (distractors). Responses are typically faster and less error-prone when responses associated to target and distractor match (e.g., HHHHH and SSSSS; congruent trials) compared with mismatch (e.g., SSHSS and HHSHH; incongruent trials; e.g., Eriksen & Schultz, 1979; Hübner & Töbel, 2019; Servant & Logan, 2019). This so-called flanker effect (and other congruency effects) are usually explained within dual-route models according to which activation produced by target and distractor features are processed along separate processing routes (Egner, 2007; Hübner et al., 2010; Kornblum et al., 1990; Ulrich et al., 2015). Congruency effects arise because these two types of activations converge at some point during task processing thereby improving (congruent trials) or worsening (incongruent trials) task performance. The visual flanker effect, for example, is attributed to insufficient attentional filtering of flankers, which leads to competing response activations in the case of incongruent trials (e.g., Mattler, 2005).

While flanker stimuli were first presented only in the visual modality, later studies have also observed robust congruency effects with other distractor modalities, such as auditory (e.g., Chan et al., 2005; Ulrich et al., 2021) or tactile flankers (e.g., Baciero et al., 2021; Wesslein et al., 2014, 2015). For example, in a recent study by Ulrich et al. (2021), robust flanker effects were observed when participants were required to respond to a central visual target (a letter H or S) while ignoring auditory flankers (i.e., spoken letters H or S). Thus, while the traditional visual flanker effect shows that visual stimulus activations cause interference during task processing, this crossmodal flanker effect demonstrates that interference can also be created by activations produced from both visual and auditory stimuli. This is in line with findings from other (non-conflict task) studies indicating that information from different input modalities can be integrated to form a coherent representation during task processing (e.g., Falchier et al., 2002; Frings & Spence, 2010; Fu et al., 2020; Spence & Ho, 2015; for a review, see Turk, 2014). Thus, with both visual and auditory distracting information, distractor-based and target-based activations superimpose to produce the observed congruency effects (e.g., Ulrich et al., 2021; Mattler, 2005). While this may suggest that at least partially shared processing operations are involved in dealing with different distractor-modalities, we might adapt our behavior exclusively in a modality-specific manner (e.g., Stephan & Koch, 2016; Yang et al., 2017). For example, it is possible that different modality-specific control mechanisms are required if the point at which distractor-based activation from visual and auditory stimuli feeds into the task-relevant visual processing stream might differ (for a similar speculation, see e.g., Ulrich et al., 2021). To shed further light on the underlying mechanisms with different distractor modalities, we investigated whether one hallmark effect reflecting global behavioral adaptation of congruency effects, the proportion congruency effect (PCE), transfers across the Eriksen task with visual and auditory flankers.

## The proportion congruency effect

Several studies have shown that the visual flanker effect (as well as other congruency effects) is larger in blocks where the proportion of congruent trials is high compared with low (for a review, see Bugg & Crump, 2012; e.g., Forster et al., 2011; Gratton et al., 1992; Jost et al., 2022; Wendt & Luna-Rodriguez, 2009). It has been proposed that this PCE essentially emerges based on two, mutually not exclusive, implementations of adaptive processing regulations. According to cognitive control accounts, processing is regulated based on higher-level cognitive control mechanisms (e.g., Egner & Hirsch, 2005; Logan & Zbrodoff, 1979). For example, target processing is enhanced and/or distractors are more strongly suppressed when distractors are more likely to produce conflict. According to memory accounts, processing is regulated based on lower-level memory traces which, for example, operate on statistical regularities without necessarily involving any higher-level cognitive control (e.g., Hommel et al., 2004; Luo et al., 2022; Mayr et al., 2003; Schmidt, 2013a, b). For example, participants learn that specific distractor-response associations appear more versus less frequently in blocks with a high- versus low-proportion of congruent trials (hereafter: high versus low PC blocks) (contingency learning, e.g., Schmidt & Besner, 2008). In the Eriksen flanker task with two letters as used in the present study (H and S), for example, the distractor letter H is more often paired with the target letter H than S in high than low PC blocks which boosts the PCE due to selective performance improvements in congruent trials in high PC blocks and in incongruent trials in low PC blocks. Several studies have provided evidence for both accounts by using list-wide PC manipulation as well as item- and tasks-specific PC manipulations (e.g., Bausenhart et al., 2021; Jacoby et al., 2003; for an overview, see Bugg & Crump, 2012).

Critically, to our knowledge, no study has examined whether the PCE obtained in trials with distractors in one modality (e.g., visual) can generally transfer to trials with distractors in another modality (e.g., auditory). To make a first step in addressing this issue, participants in the present study were required to always respond to a central visual target (H or S) as in the classical Eriksen flanker task (Eriksen & Eriksen, 1974) and the flankers were either visual (e.g., HSH) or auditory (e.g., spoken letters presented via headphones or loudspeakers). As is elaborated in more detail later, after we established whether a PCE can be observed in this bimodal auditory-visual flanker paradigm, we selectively applied a PC manipulation to one of the two flanker modalities (i.e., inducer distractor modality with high versus low PC) to investigate the potential transfer to the other modality (i.e., diagnostic distractor modality with equal PC).

Although the present study was not designed to contrast cognitive control versus memory accounts underlying the PCE—and instead to provide a strong test whether the PCE obtained in trials with distractors in one modality (e.g., visual) can generally transfer to trials with distractors in another modality (e.g., auditory)—the results can also help advance theorizing along these accounts.^2^ According to cognitive control accounts, there could be transfer of the PCE across trials with different distractor modalities if cognitive control strengthens (visual) target processing and/or suppresses (visual or auditory) distractors in an amodal manner (e.g., Botvinick et al., 2001). Consider also that dual-process conflict-task models often (at least implicitly) assume that postperceptual—and hence presumably abstract (amodal)—activations are superimposed during decision-making (e.g., Botvinick et al., 2001; De Jong et al., 1994; Eimer et al., 1995; Ulrich et al., 2015). According to memory accounts (e.g., contingency learning), there could also be transfer of the PCE across trials with different distractor modalities if participants learn, at least partially, the pairing of specific distractor-response links in an amodal manner (e.g., the modality-unspecific identity of the distractor letter H is more often paired with the target letter H in high PC blocks). Thus, there are good reasons to assume that we will observe global behavioral adaptation effects with different distractor modalities when the target remains visual. On the other hand, clear differences in processing auditory versus visual stimuli (e.g., Bendixen et al., 2010) might also result in distractor modality-specific global behavioral adaptations (e.g., learning from modality-specific stimulus–response links).

It seems difficult to predict the outcome of this study when considering prior studies with PC manipulations to visual tasks. For example, Bausenhart et al. (2021) intermixed the standard visual Eriksen task with a standard visual Simon task (i.e., location as distractor) and investigated transfer of the inducer conflict task (with high versus low PC) to the diagnostic conflict task (with equal PC). While they replicated standard PCE for the inducer task, no transfer effects were found in diagnostic tasks. Considering that they used the same targets for both tasks (i.e., H versus S), it seems that the underlying processing mechanisms of a PC manipulation are restricted to the specific type of distractors (i.e., location in the Simon task or identity of flankers in the Eriksen task) or to the specific target location (i.e., left versus right in the Simon task or central in the Eriksen task). On the other hand, there are also some hints for distractor-general effects with other combinations of conflict tasks (e.g., Funes et al., 2010; Wühr et al., 2015). For example, Funes et al. (2010) used a similar approach as Bausenhart et al. (2021), but they intermixed a spatial Stroop with a Simon task and conflict in both cases was based on the location of the target (up/down: Stroop and left/right: Simon). Thus, this study indicates that transfer effects could at least partially be observed when tasks are similar in terms of both targets and the type of distracting information (here: spatial information). Considering that in our study the targets were always centrally presented, and the different distractor modalities relied on similar informational input (i.e., letter identity H and S), it seems possible to see transfer effects with different distractor modalities. On the other hand, any mechanisms underlying global behavioral adaptation effects might be restricted to both the specific identities and modalities of distractors (i.e., auditory or visual).

Given the lack of prior research investigating global behavioral adaption with different distractor modalities, it is particularly reasonable to consider studies investigating a marker of local behavioral adaptation, the congruency sequence effect (CSE). The CSE indicates that congruency effects are smaller when the previous trial was incongruent compared with when it was congruent (e.g., Gratton et al., 1992; Rey-Mermet et al., 2019; Weissman et al., 2014; Wendt et al., 2006; Yang et al., 2021). This effect can be explained by both higher- (i.e., cognitive control) and lower-level (e.g., contingency learning) accounts. Regardless of the specific account(s) underlying the CSE, it seems particularly interesting that both modality and task representations can act as crucial boundary conditions of such local behavioral adaptation effects (e.g., Grant et al., 2020; Hazeltine et al., 2011; Kreutzfeldt et al., 2016; Yang et al., 2017). In essence, it seems that CSEs can only be observed with different target and/or distractor modalities when additional changes in the task structure help make the modality difference less distinct (i.e., see also Grant et al., 2020; Grant & Weissman, 2022). We therefore opted to only vary the modality (visual or auditory) of distractors while keeping the target modality constant to provide a strong first test of whether the PCE can in principle be observed across different distractor modalities. Moreover, the properties of distractors were kept similar (i.e., letters), because it seems possible that differences in distractor properties may also contribute to building different task structures (but see Yang et al., 2017, for a modality-specific CSE while accounting for distinct distractor modalities).^3^ Finally, it should also be emphasized that it is not clear whether findings and implications of studies investigating CSEs are readily transferable to PCEs. While there could be certainly common mechanisms underlying PCE and CSE,^4^ there could also be at least partially distinct mechanisms (e.g., Funes et al., 2010; Wühr et al., 2015). We will return to this issue in our General Discussion where we also refer to the exploratory findings of additional analyses examining CSEs, but for now we focus on the investigation of the PCE as a function of different distractor modalities.

## Overview of the present experiments

In Experiment 1, we investigated whether the PCE can be generally observed in a bimodal auditory-visual Flanker paradigm with a central visual target and auditory or visual flankers. Specifically, we manipulated the proportion of congruent and incongruent trials across the two flanker modalities (i.e., in half of the blocks 80% congruent and in the other 20% congruent). In Experiments 2A and 2B, the PC manipulation was selectively applied to either the visual (Experiment 2A) or auditory (Experiment 2B) flanker modality and hence one modality served as the inducer-modality (i.e., in half of the blocks 83% and in the other 17% congruent trials) and the other as the diagnostic-modality (i.e., 50% congruent in each block).^5^ Thus, we used a similar approach as others investigating the transfer of a PC manipulation across different visual tasks (e.g., Bausenhart et al., 2021; Shichel & Goldfarb, 2022; Wühr et al., 2015). The key question is whether a PCE in the distractor inducer-modality would be also observed in the distractor diagnostic-modality for which the proportion of congruent/incongruent trials was equal. If global behavioral adaptations transfer across different distractor modalities, the congruency effect should be reduced in blocks with a high proportion of incongruent compared with congruent trials not only for distractor inducer but also for distractor diagnostic-modality trials. Alternatively, this effect should be only seen for trials in the inducer-but not the diagnostic-modality when there is no transfer.

In addition to the main analyses at the level of mean reaction time (RT) and mean error rate (ER), we also conducted distributional analyses to understand better the influence of different distractor modalities on conflict processing. Specifically, we constructed RT delta plots for each condition (i.e., inducer/diagnostic distractor modality X high/low PC) to illustrate the congruency effects across the RT distribution (e.g., Heuer et al., 2023). Some previous studies using a PC manipulation in a visual Simon task have shown that the delta plots of low PC blocks are generally below the delta plots of high PC blocks across the entire RT distribution suggesting that global behavioral adjustments can be observed for both fast and slow responses, for instance, by adjusting the strength of distractor to target processing throughout a trial (e.g., Hübner & Töbel, 2019; Ridderinkhof et al., 2004; Ridderinkhof, 2002). Critically, these studies also showed a larger negative slope for low compared with high PC delta plots. This could suggest that the underlying mechanisms induced by the PC manipulation also influences the timing of target to distractor processing. Thus, the delta plot analyses of the present study could reveal additional insights into the underlying mechanisms when adapting behavior to a global proportion congruency manipulation in the Eriksen flanker task with visual and auditory distractors.

Finally, we also examined local behavioral adaptations, specifically the CSE (e.g., Gratton et al., 1992). These exploratory analyses are reported in Appendix Fig. 8, 9 and 10 and are further discussed in our General Discussion.

## Experiment 1

In the first experiment, we investigated whether a proportion congruency (PC) manipulation applied to both distractor modalities (i.e., visual and auditory) would modulate the congruency effect in the Eriksen flanker task with visual targets. Thus, this also allowed us to see whether auditory-based congruency effects are smaller than visual-based congruency effects and whether the effect of the proportion manipulation differs across distractor types.

### Method

#### Participants

Overall, 50 participants were tested, but data of five participants were excluded due to low performance (see data preparation procedure). The remaining 45 participants (34 female, 37 right-handed) age ranged from 18 to 45 years (*M*_age_ = 21.91 years). Participants were recruited via advertisements on the campus of the University of Tübingen, social media and internal departmental e-mail lists and received course credits. In this and in the following experiments, all participants provided informed consent before testing. Furthermore, all experiments were in accordance with the ethical standards set by the local ethics committee and with the 1964 Helsinki Declaration and its later amendments or comparable ethical standards.

#### Sample size justification

The sample size of 50 participants in Experiment 1 was somewhat arbitrarily yet conservatively set.^6^ While the size of the observed PCE in Experiment 1 was very large (η_p_^2^ = .84 across distractor modalities), we reasoned that possible transfer effects for the distractor modality without a PC manipulation would be smaller. Thus, as can be seen in our preregistration, we opted to continue with a large sample size of *N* = 60 in both Experiment 2A and 2B. With this sample size, we would have over 80% power to detect a significant PCE of at least η_p_^2^ = .12 at a significance level of α = .05.

#### Apparatus and stimuli

The experiment was conducted online using the JavaScript library jsPsych (De Leeuw, 2015). All visual stimuli were presented on a grey background, and auditory stimuli were presented via headphones or speakers. A centrally positioned plus sign served as the fixation point. Target and distractor (flanker) stimuli were the letters H and S. For each participant, the two stimulus letters were randomly assigned to left- and right-hand response keys. Target stimuli were always visually presented in the center of the screen. Visual flankers were the letters H or S presented on each side of the target stimulus (e.g., HSH) and auditory flankers were the spoken letters H or S (duration of 500 ms) presented by a male voice in German (similar to Ulrich et al., 2021, auditory stimuli were obtained from https://freesound.org/people/reinsamba/sounds/69247/). In catch trials, the letter X was presented instead of the target letter and participants had to respond to the (visual or auditory) flankers. These catch trials were implemented to ensure the proper functioning of the sound system^7^. Responses were made by pressing the Q- or P-key with the left or right index-fingers, respectively.

#### Procedure

In total, there were 12 experimental blocks with 84 trials in each block equally distributed to the visual and auditory distractor modality, with four of them being catch trials (Table 1). For half of the participants, the first six blocks had a high PC and the other half a low PC, whereas this order was revered for half of the participants. In the high PC blocks 76.19% of all trials were congruent, whereas in the low PC blocks only 23.81% of the trials were congruent. Excluding the catch trials, the ratio was 80% versus 20% congruent trials in a block.

**Table 1:** Overview of the number of trials for each distractor Modality and Congruency in high and low PC Blocks in Experiment 1

Modality	Congruency	*N* high PC Block	N low PC Block
Auditory	Congruent	32	8
Auditory	Incongruent	8	32
Visual	Congruent	32	8
Visual	Incongruent	8	32
Auditory	Catch	2	2
Visual	Catch	2	2
Total		84	84
Overall PC		76.19%	23.81%
Non-Catch trial PC		80%	20%

Overview of the number of trials (*N*) as a function of distractor modality (auditory or visual) and congruency separately for high- and low-proportion congruency (PC) blocks. The last two rows show the percentage of congruent trials calculated with (overall PC) and without catch trials (Non-Catch trial PC)

**Fig. 1:**
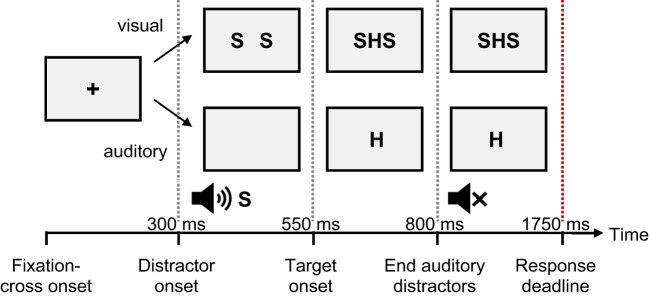
Schematic depiction of possible trial sequences

Figure 1 displays a possible trial sequence. Each trial started with the presentation of a fixation cross in the center of the screen for 300 ms, after which the auditory or visual distractors were added. Because the congruency effect with auditory distractors was most pronounced with 250 ms delay of visual targets (cf. Ulrich et al., 2021), we opted for this delay. After 250 ms, the visual target stimulus was presented at the center of the screen. Note that auditory distractors (i.e., one spoken letter H) were present for another 250 ms, whereas visual distractors remained on the screen (e.g., HHH) until participants responded up to a response deadline of 1,750 ms. Thus, we chose to use the standard versions of the flanker task with auditory and visual distractors, resulting in different distractor presentation times. We reasoned that presenting several auditory flankers until participants responded might lead to the emergence of additional strategies (e.g., participants waiting with responding until a second or third letter is spoken). Furthermore, we reasoned that the longer, response-dependent presentation times of visual flankers should not have an impact, as previous research has shown that distractor processing remains unaffected by presentation times in the visual Simon task (Ellinghaus et al., 2018) and visual flanker task (Ellinghaus et al., 2023).

After correct responses, the next trial started after an intertrial interval (ITI) of 1 s. After incorrect responses, an additional feedback screen was presented for 2 s before the ITI indicating in German whether a wrong response was made (“wrong!”) or no response was made with the response deadline (“too slow”).

Participants were instructed to respond to the target stimuli when an H or S was presented and to ignore the flanker. However, they were instructed to respond to the (auditory or visual) flankers when a X instead of a target letter appeared. After each block participants could take a self-paced break.

#### Data preparation

The first block in each PC condition was considered practice and excluded from any analyses (Blocks 1 and 7). Following this, data of participants with overall accuracy of less than 80% (three participants) were excluded. We additionally excluded two participants with an overall error rate of more than 45%^8^ in catch trials. For all reported RT and ER analyses, we additionally excluded catch trials, trials following catch trials, the first trial of each block, trials with too fast responses (<100 ms) (0.53%), and trials without any response (0.14%). For RT analyses, only correct trials were used. The majority of participants across all experiments had a minimum of 12 trials per condition for RT analyses, considering only correct trials, which ensured reasonable RT estimates. When excluding the two participants with five and eight trials in a specific condition, the results remained virtually similar.

#### Design

Following Bausenhart et al. (2021), individual RT- and ER-based congruency effects (i.e., RT_incongruent_ − RT_congruent_; ER_incongruent_ − ER_congruent_) were calculated separately for each participant within each condition (cf. Bausenhart et al., 2021; see Appendix Table 3, 4, 5, 6, 7 and 8 for the results of mean RT and mean PE).^9^ Following this, two-factorial repeated-measures analyses of variance (ANOVAs), with PC (high versus low) and current flanker modality (auditory versus visual) as independent variables and congruency effects in either RT or ER as dependent variables, were computed (with follow-up *t* tests for pairwise comparisons).

For the delta plot analyses, we used the R package DMCfun (Mackenzie & Dudschig, 2021). Specifically, we binned the rank-ordered RTs into five RT percentiles separately for each participant within each of the four conditions (i.e., low/high PC and auditory/visual flankers). Similar results were obtained in delta plot analyses using alternative numbers of percentiles (e.g., 3 or 8). We then summarized the delta plot for each participant and condition with a linear regression fit (e.g., Ellinghaus et al., 2018; Gade et al., 2020; Mittelstädt & Miller, 2018; Mittelstädt et al., 2022a, b, c; Pratte et al., 2010) and compared the slopes with a two-factorial repeated-measures ANOVA.

### Results and discussion

#### Mean RT and PE

**Fig. 2:**
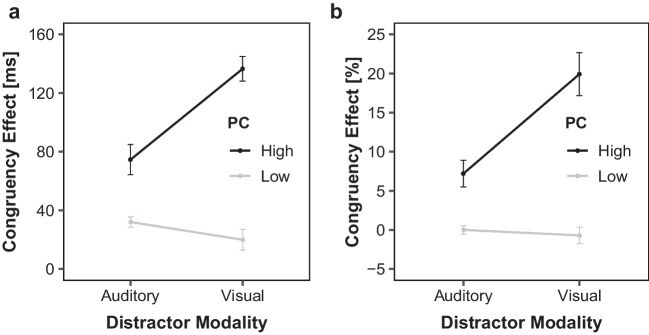
Current Distractor Modality and PC dependent Congruency Effects. Mean congruency effects in reaction times (**a**) and mean percentage error (**b**) of Experiment 1 as a function of current distractor modality (auditory, visual) and proportion congruency (PC; low, high). Error bars indicate 1 standard error (*SE*) of the corresponding means

Figure 2a illustrates mean congruency RT effects as a function of PC and current distractor modality condition. The ANOVA revealed a significant main effect of PC reflecting larger congruency effects in high compared with low PC blocks (106 ms versus 26 ms), *F*(1, 44) = 238.09, *p* < .001, η_p_^2^ = 0.84. Furthermore, there was a main effect of distractor modality indicating more pronounced congruency effects with visual compared with auditory distractors (78 ms versus 53 ms), *F*(1, 44) = 11.87, *p* < .001, η_p_^2^ = 0.21. Finally, there was an interaction between distractor modality and PC, *F*(1, 44) = 46.57, *p* < .001, η_p_^2^ = 0.51. As is visible in Fig. 2a, the PCE was present for both distractor modalities (both *p*s < .001), but larger with visual (136 − 20 = 116 ms) than auditory distractors (75 − 32 = 43 ms).

Figure 2b shows the corresponding ER-based congruency effects. There was again a main effect of PC, reflecting larger congruency ER effects in high compared with low PC blocks (13.6% versus −0.3%), *F*(1, 44) = 50.19, *p* < .001, η_p_^2^ = 0.53. There also was a main effect of distractor modality, *F*(1, 44) = 17.00, *p* < .001, η_p_^2^ = 0.28. Congruency effects were larger with visual (9.6%) than auditory (3.6%) distractors. Finally, the interaction was also significant, *F*(1, 44) = 46.85, *p* < .001, η_p_^2^ = 0.52. Mirroring the RT results, there were significant PCEs for both distractor modalities (both *p*s < .001), but this effect was larger for visual (19.9 − (−0.7) = 20.6%) compared with auditory distractor trials (7.2 − 0.0 = 7.2%).

#### Delta plots

**Fig. 3:**
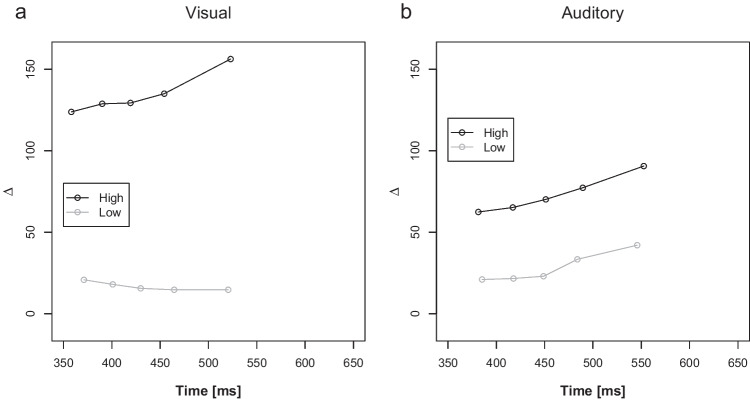
Distractor Modality specific PC dependent Delta plots. Delta plots showing incongruent minus congruent RT differences (∆ in ms) within each of five RT bins plotted against the bin average (Time in ms) as a function of proportion of congruency (PC): low and high, separately for visual (**a**) and auditory (**b**) distractor modalities

Figure 3 illustrates the delta plots as a function of low and high PC blocks and current distractor modality. The ANOVA on mean slopes only revealed a significant interaction between PC and distractor modality, *F*(1, 44) = 10.06, *p* = .003, η_p_^2^ = 0.19. Pairwise comparison revealed no significant difference between low and high PC slopes for auditory distractors (0.16 versus 0.11, *p* = .288; Fig. 3a), but significantly different slopes for low and high PC blocks for visual distractors (−0.04 versus 0.17, *p* = .001; Fig. 3b).

In sum, using a hybrid Eriksen flanker paradigm with visual and auditory distractors, the congruency effects were smaller in low compared with high PC blocks for both distractor modality types. This modulation was larger for visual than auditory distractors—presumably due to the overall larger congruency effects within this modality. The delta plots suggest that the effects of the PC modulation were present throughout the entire RT distribution (and hence for faster and slower responses). Interestingly, the slope of delta plots similarly increased across the PC conditions for auditory distractors but was less increasing (and even decreasing for the low PC condition) for visual distractors. These distinct delta plots slopes demonstrate distractor modality-specific global behavioral adaptations. One might speculate that the slopes reflect the time-course of distractor activation (e.g., Ellinghaus et al., 2018; Ellinghaus & Miller, 2018; Mackenzie et al., 2022; Ridderinkhof, 2002). Accordingly, these results may suggest that the PC modulation with visual distractors induce biases in both the strength and timing of target-to-distractor processing, whereas with auditory distractors only the strength is adjusted (cf. Mittelstädt et al., 2022c).

## Experiment 2A and 2B

Experiment 1 indicated that a blockwise PC manipulation applied to trials with auditory and visual flankers can elicit global behavioral adaptation effects across both distractor modalities. In the following two experiments, we investigate whether these behavioral effects occur in purely distractor modality-specific ways or not. For this purpose, the PC manipulation was selectively implemented to either the visual (Experiment 2A) or auditory (Experiment 2B) flanker modality. Thus, one distractor modality served as inducer-modality and the other as diagnostic-modality, as the PC was not manipulated in the latter.

### Method

#### Participants

In both Experiment 2A and Experiment 2B, participants were tested online and were recruited via Prolific or from the same pool as in Experiment 1. Overall, 60 participants (28 female, 55 right-handed, *M*_age_ = 28.78 ranging from 19 to 70) were tested in Experiment 2A and 60 participants (24 female, 53 right-handed, *M*_age_ = 27.72 ranging from 18 to 59) were tested in Experiment 2B. Preregistrations are available via the Open Science Framework (OSF) at https://osf.io/8d3vn and https://osf.io/vkzgr, respectively.

#### Stimulus, apparatus, and procedure

The method was the same as in Experiment 1 except otherwise described. In contrast to Experiment 1, the PC manipulation was only applied to either the visual or auditory distractor modality while keeping the proportion of congruent trials similar in the other distractor modality. In Experiment 2A, the visual distractor modality was the inducer modality, and the auditory distractor modality was the diagnostic modality, whereas this was reversed for Experiment 2B. As can be seen from Table 2, the PC only differed for the inducer but not diagnostic trials. In order to maximize the number of diagnostic trials for the exploratory CSE analyses (reported in Appendix Figs. 8, 9 and 10), catch trials were never presented before diagnostic trials to ensure reliable RT and ER estimates. As mentioned in the data preparation section, trials following catch trials were excluded when analyzing both the PCE and CSE. Therefore, although catch trials could predict the distractor modality in the subsequent trials, this association should not have any impact as these trials were excluded from all analyses reported in the main text and in the Appendices. Note also that the results were similar even when including these excluded trials.

**Table 2:** Overview of the number of trials for each distractor Modality and Congruency in high and low PC Blocks in Experiments 2A and B

Modality	Congruency	*N* high PC Block	*N* low PC Block
Diagnostic	Congruent	12	12
Diagnostic	Incongruent	12	12
Inducer	Congruent	60	12
Inducer	Incongruent	12	60
Auditory	Catch	2	2
Visual	Catch	2	2
Total		100	100
PC inducer		83%	17%
PC diagnostic		50%	50%
Overall Non-Catch trial PC		75%	25%

Overview of the number of trials (*N*) as a function of distractor modality (inducer or diagnostic) and congruency separately for high and low proportion congruency (PC) blocks. The last three rows show the percentage of congruent trials calculated with in either inducer or diagnostic conditions and overall PC (with inducer and diagnostic trials) without catch trials

#### Data preparation

Data preparation was the same as in Experiment 1. Following our preregistration, in Experiment 2A, the data of eight participants had to be excluded due to overall error rates of more than 20% and/or error rates in catch trials of more than 45%. In Experiment 2B, the data of two participants had to be excluded (due to >45% catch trial error rate). From the data of the remaining participants, we again excluded trials with no responses (Experiment 2A: 0.18%, Experiment 2B: 0.37%) and too-fast responses (Experiment 2A: 0.10%, Experiment 2B: 0.12%).

#### Design

Two factorial repeated measure ANOVAs with RT- and ER-based congruency effects as the dependent variables and PC (low, high) and distractor modality (auditory, visual) as independent variables were computed. Delta plots were computed and analyzed in an analogous manner as in Experiment 1. Note that in Experiment 2A, the visual (auditory) distractor modality was the inducer (diagnostic) distractor modality, whereas in Experiment 2B, the auditory (visual) distractor modality was the inducer (diagnostic) distractor modality.

### Results and discussion Experiment 2A

Similar to Experiment 1, congruency effects are based on the RT and PE. In Experiment 2A, the inducer distractor modality was visual, so the proportion of congruency was only manipulated in visual trials.

#### Mean RT and PE

**Fig. 4:**
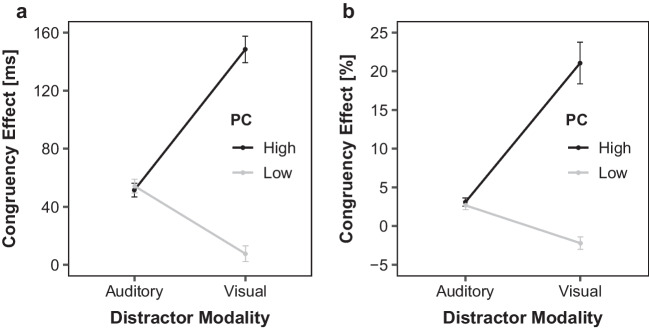
Current Distractor Modality and PC dependent Congruency Effects for visual Inducer. Mean congruency effects in reaction times (**a**) and mean percentage error (**b**) of Experiment 2A as a function of current distractor modality (auditory, visual) and proportion congruency (PC; low, high). Error bars indicate 1 standard error (*SE*) of the corresponding means

Figure 4 illustrates the mean RT- and ER-based congruency effects as a function of PC and distractor modality. There was a significant main effect of PC reflecting larger congruency effects in blocks with high compared with a low PC (100 ms versus 31 ms), *F*(1, 51) = 166.13, *p* < .001, η_p_^2^ = 0.77. Furthermore, there was a main effect of distractor modality indicating more pronounced congruency effects with visual (inducer) compared with auditory (diagnostic) distractors (78 ms versus 53 ms), *F*(1, 51) = 18.26, *p* < .001, η_p_^2^ = 0.26. Finally, there was an interaction between distractor modality and PC, *F*(1, 51) = 290.28, *p* < .001, η_p_^2^ = 0.85. As is visible in Fig. 4a, the PCE was only present in the visual inducer distractor modality (148 ms versus 8 ms; *p* < .001), but not in the auditory, diagnostic distractor modality (51 ms versus 54 ms; *p* = .596).

Figure 4b shows the corresponding ER-based congruency effects. Again, there was a main effect of PC, reflecting larger ER-based congruency effects in high compared with low PC blocks (12.1% versus 0.2%), *F*(1, 51) = 65.77, *p* < .001, η_p_^2^ = 0.56. Similar to the RT-based congruency effects, there was a main effect of distractor modality, *F*(1, 51) = 24.78, *p* < .001, η_p_^2^ = 0.33. with larger congruency effects in inducer (visual) than diagnostic (auditory) trials (9.4% versus 2.9%). Finally, the interaction between distractor modality and PC was also significant, *F*(1, 51) = 62.42, *p* < .001, η_p_^2^ = 0.55. There were significant PCEs in trials with visual inducer distractors (21.1% versus −2.2%; *p* < .001), but not with auditory diagnostic distractors (3.1% versus 2.7%; *p* = .485).

#### Delta plots

**Fig. 5:**
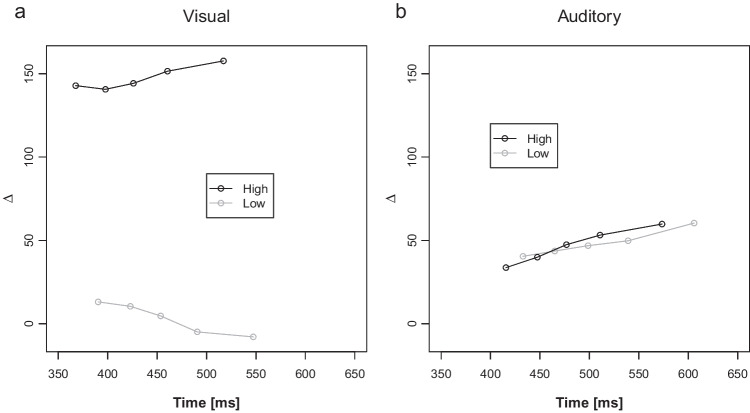
Distractor Modality specific and PC dependent Delta plots for visual Inducer (Experiment 2A). Delta plots showing incongruent minus congruent RT differences within each of 5 RT bins plotted against the bin average (Time in ms) as a function of proportion of congruency (PC): low = black and high = grey, separately for visual (**a**) and auditory (**b**) distractor modalities. Inducer modality was visual

Figure 5 illustrates the delta plots as a function of low and high PC blocks and current distractor modality. The ANOVA on mean slopes revealed a significant main effect of PC, *F*(1, 51) = 17.10, *p* < .001, η_p_^2^ = 0.23, reflecting a more negative-going slope in low PC (−0.03) compared with high PC blocks (0.13). Additionally, there was a main effect of distractor modality, *F*(1, 51) = 43.22, *p* < .001, η_p_^2^ = 0.46, indicating more negative-going delta plots in visual-inducer trials compared with auditory-diagnostic trials (−0.03 versus 0.14). The interaction between PC and distractor modality, was also significant *F*(1, 51) = 10.09, *p* = .003, η_p_^2^ = 0.17. Pairwise comparison revealed no significant difference between low and high PC slopes for auditory distractors (0.17 versus 0.13; *p* = .227), but similar to Experiment 1, significantly more negative-going slopes for low than high PC blocks for visual distractors (−0.17 versus 0.01; *p* < .001).

### Results and discussion Experiment 2B

Similar to Experiment 1 and 2A, congruency effects are based on the RT and PE. In Experiment 2B the inducer distractor modality was auditory, so the proportion of congruency was only manipulated in auditory distractor trials and not in visual ones.

#### Mean RT and PE

**Fig. 6:**
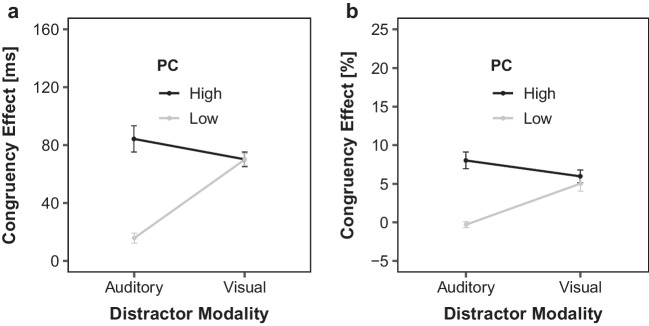
Current Distractor Modality and PC dependent Congruency Effects for auditory Inducer. Mean congruency effects in reaction times (**a**) and mean percentage error (**b**) of Experiment 2B as a function of current distractor modality (auditory, visual) and proportion congruency (PC; low, high). Error bars indicate 1 standard error (*SE*) of the corresponding means

Figure 6 shows the corresponding mean RT- and ER-based congruency effect pattern as a function of PC and current distractor modality condition. Similar to Experiment 2A, there was a significant main effect of PC reflecting larger congruency effects in blocks with high compared with low PCs (77 ms versus 42 ms), *F*(1, 57) = 46.47, *p* < .001, η_p_^2^ = 0.45. The significant main effect of distractor modality indicated larger congruency effects with visual-diagnostic compared with auditory-inducer distractors (70 ms versus 50 ms), *F*(1, 57) = 10.56, *p* = .002, η_p_^2^ = 0.16. Lastly, there once again was an interaction between distractor modality and PC, *F*(1, 57) = 58.85, *p* < .001, η_p_^2^ = 0.51. As is visible in Fig. 6a, the RT-based PCE was only present in the auditory distractor (inducer) (84 ms versus 16 ms; *p* < .001), but not in the visual (diagnostic) one (both 70 ms;* p* = .978).

Figure 6b depicts the corresponding ER-based congruency effects. There was a significant main effect of PC, with larger error-congruency effects in high compared with low PC blocks (7.0% versus 2.4%), *F*(1, 57) = 51.21, *p* < .001, η_p_^2^ = 0.47. The main effect of distractor modality was only marginal significant, *F*(1, 57) = 3.20, *p* = .079, η_p_^2^ = 0.05. As in the previous experiment, there was a significant interaction between distractor modality and PC *F*(1, 57) = 19.32, *p* < .001, η_p_^2^ = 0.25. The PCE was only significant in trials with auditory-diagnostic distractors (8.0% versus −0.3%; *p* < .001), but not with visual-inducer distractors (6.0% versus 5.0%; *p* = .347). Thus, as in Experiment 2A, it seems that global processing adjustments act in a distractor modality-specific way.

#### Delta plots

**Fig. 7:**
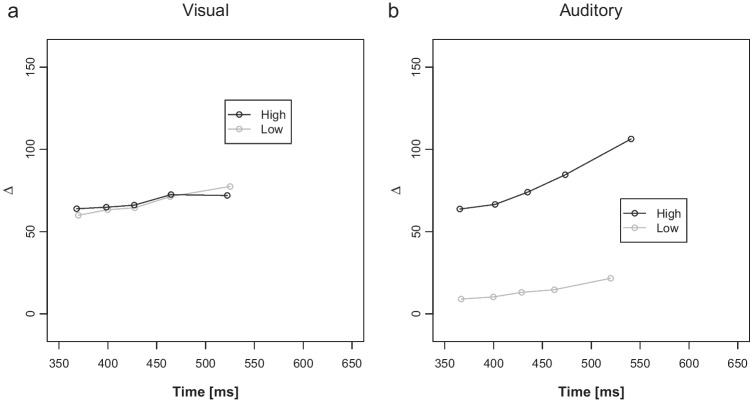
Distractor Modality specific and PC dependent Delta plots for auditory Inducer (Experiment 2B). Delta plots showing incongruent minus congruent RT differences within each of 5 RT bins plotted against the bin average (Time in ms) as a function of proportion of congruency (PC): low = black and high = grey, separately for visual (**a**) and auditory (**b**) distractor modalities. Inducer modality was auditory

Figure 7 illustrates the delta plots. The ANOVA on mean slopes revealed no significant main effects or interactions *F*(1, 57) < 2.64, *p*s > .110, η_p_^2^s < 0.04. To make the results more comparable to Experiment 2A, pairwise comparisons were conducted for each distractor modality. There was neither a significant difference between low and high PC slopes for auditory distractors (0.098 versus 0.163; *p* = .228), nor for visual distractors (0.107 versus 0.063; *p* = .339).

## General discussion

In the present study, we contrasted modality-specificity versus -generality of global behavioral adaptations to interference arising from different distractor modalities. Specifically, a series of experiments were conducted using an Eriksen flanker task in which the target was always visual but the distractor was either auditory (Ulrich et al., 2021) or visual (Eriksen & Eriksen, 1974). In Experiment 1, a PC manipulation was applied for both distractor modalities, whereas in Experiments 2A and 2B, this manipulation was only applied to one inducer distractor modality to test potential transfer of behavioral adaptations to the other diagnostic distractor modality. Across all experiments, there was evidence for global behavioral adaptations as reflected in substantial PCEs for distractor modalities with a PC manipulation (Experiment 1: visual and auditory, Experiment 2A: visual; Experiment 2B: auditory). Critically, there was no evidence of transfer to the diagnostic distractor modalities in Experiments 2A and 2B: The behavioral adaptation due to a proportion congruency manipulation were specific to the type of distractor modality.^10^

To begin with, the present study extends previous findings of global behavioral adaptation effects (as reflected in PCEs) on interference within the visual modality (for a review, see Bugg & Crump, 2012; e.g., Shichel & Goldfarb, 2022; Thomson et al., 2014) to interference across the visual and auditory modalities. Thus, this demonstrates that the recently observed crossmodal flanker effect by Ulrich et al. (2021) can be qualitatively modulated by similar manipulations as visual flanker effects. Not surprisingly, the congruency effects were generally larger with visual than auditory distractors. Thus, similar to others (e.g., Frings & Spence, 2010), this indicates that visual target processing is more prone to interference within the visual modality than it is to crossmodal processing. Furthermore, one might speculate that vision might generally dominate audition which would lead to larger interference from visual than auditory distractors (cf., ventriloquist effect and visual dominance, e.g., Bendixen et al., 2010; Bresciani et al., 2008; see also Kreutzfeldt et al., 2015; Lukas et al., 2010, for further evidence of attention-based visual dominance).

Critically, while our results showed strong evidence for global behavioral adaptations with both distractor modalities, there was no evidence for the transfer of PCEs from one to the other distractor modality in Experiments 2A and 2B. As mentioned in the introduction, it is unclear whether in the present study the observed behavioral adaptations reflect higher level cognitive control and/or lower-level memory-based processes such as contingency learning.^11^ The novel empirical demonstration that PCEs emerge in a distractor modality-specific manner does not help to distinguish between cognitive control and memory accounts. However, in addition to its empirical contributions (see also the delta plot section below), the study’s findings also have some theoretical implications for these accounts.

### Implications for cognitive control and memory accounts

First, it seems difficult to explain our findings by exclusively relying on higher-level cognitive control accounts which—at least implicitly—assume that the PCE is due to modality-independent target amplification and/or distractor processing (e.g., Botvinick et al., 2001; Logan & Zbrodoff, 1979; Ulrich et al., 2015; Wühr et al., 2015). These accounts would require some elaboration to account for the distractor modality-specific effects. Considering the targets were always visually presented and only distractor modalities varied, target amplification would have been already sufficient to observe transfer across the different distractor modality trials. Thus, assuming that the PCE in the present set-up arises (at least partially) due to cognitive control, it seems more likely that distractor-suppression involves modality-specific control mechanisms. A related, nonmutually exclusive possibility is that auditory distractors may elicit somewhat different conflict compared with visual distractors when responding to visual targets and require modality-specific global control mechanisms.

Second, our findings could also be interpreted with the framework of memory accounts. These accounts essentially propose that processing is regulated based on memory traces that operate on statistical regularities (e.g., contingency learning) without necessarily involving top-down cognitive control (e.g., Hommel, 2004; Mayr et al., 2003; Schmidt, 2013a, b; Schmidt & Besner, 2008). However, even if we assume that the global behavioral adaptation effects in the present study arise due to memory-based processes such as contingency learning, the results provide novel implications. For example, based on contingency learning accounts, it would have been possible to observe transfer if participants would have learned that the specific (amodal) letter identities of distractor are differentially paired with the target letter in high versus low PC blocks. However, it seems that these accounts can only explain the present findings by assuming the learning of modality-specific distractor–target associations. Similarly, the results also help to constrain theorizing within other memory accounts than contingency learning. For example, the temporal learning account proposed by Schmidt et al. (2013b) suggests that the cognitive system learns how and when to respond throughout the experiment. When the list-wide PC is low, the expected response time is more similar to the mean RT in incongruent trials than when the list-wide PC is high. Consequently, the transfer to diagnostic items in previous PC studies may reflect temporal learning (e.g., Schmidt, 2013a; but see Cohen-Shikora et al., 2019, for evidence against this notion). However, as we did not observe any transfer effects to diagnostic items in a different distractor modality in our study, these results might only be reconciled with the temporal learning account if we posit the existence of modality-specific, time-based memory traces.

Of course, it is also possible to interpret the present results using integrative accounts that propose memory-based representations, such as event files, incorporating stimulus- and response-specific codes, as well as more abstract control codes (e.g., Frings et al., 2020; Jiang et al., 2015; Schumacher & Hazeltine, 2016; Verguts & Notebaert, 2009). Within these theoretical frameworks, the findings of the present study suggest an association between specific distractor modalities and modality-specific control codes that are generated based on conflict proportion. In sum, the present results have implications for advancing theorizing within memory-based, control-based, and combined accounts of conflict processing. Understanding how to conceptualize the impact of different distractor modalities within these accounts is particularly valuable because, as stated in the introduction, to the best of our knowledge, no prior studies have examined the impact of a PC manipulation using different distractor modalities.

As a first step, we therefore intentionally selected the present paradigm and chose not to control for memory processes such as the learning of distractor-response contingencies (see, e.g., Bausenhart et al., 2021; Shichel & Goldfarb, 2022, for similar paradigms with a proportion congruency manipulation using only visual distractors). Our reasoning was that this approach would increase the likelihood of detecting any potential transfer effects. Naturally, it is yet to be determined whether the findings of our study generalize to other paradigms that utilize a proportion congruency manipulation (e.g., Braem et al., 2019; Sprengel et al., 2022). For example, it seems possible that with larger stimulus sets, the involvement of higher-order, potentially amodal, cognitive control processes increases as the opportunity for contingency learning decreases (cf. Bugg, 2014), which may enable observing transfer effects across different distractor modalities. Furthermore, it remains to be determined whether the findings will extend to situations where a PC manipulation is applied to visual or auditory distractors while participants respond to auditory target stimuli. The current findings strongly indicate that the observed behavioral adaptations with visual targets were specific to the modality of the distractors. Consequently, it is reasonable to anticipate similar outcomes with auditory targets, but this is of course an empirical question that requires further investigation.

### Delta plots and their implications

When theorizing about the underlying mechanisms of congruency effects, it also seems useful to go beyond mean RTs and examine delta plots. First, the present delta plots indicate that PCEs were substantial throughout the RT distribution, suggesting that the mechanisms producing global behavioral adaptation effects on a mean level are present independent from response speed. Furthermore, the overlapping delta plots in diagnostic trials illustrate that there was no evidence for transfer across distractor modalities even when examining the entire distribution.^12^ Therefore, the delta plot pattern reinforces the modality-specific behavioral adaptation to the PC manipulation observed at the mean RT level. This is significant because relying solely on mean RTs can make it challenging to discern whether similar or different conflict effects across conditions can be confounded by time-dependent distractor processing (e.g., Mittelstädt & Miller, 2020; Mittelstädt et al., 2022a, b, c). For example, in our study, processing of auditory distractors might have been quicker than visual distractors (cf. Jain et al., 2015), which could have resulted in potential modulations for auditory distractors being primarily present for faster responses. Additionally, target processing was generally faster with visual stimuli compared with distractor processing (see Appendix Table 3, 4, 5, 6, 7 and 8, Experiment 1, where both distractor modalities appeared equally often). Consequently, time-varying fluctuations in distractor processing could potentially influence mean congruency effects simply due to changes in overall target processing speed (e.g., Mittelstädt et al., 2022a, b, c; Mittelstädt & Miller, 2020).

Second, similar to others (e.g., Hübner & Töbel, 2019; Luo et al., 2022), the slopes of delta plots with visual distractors (Experiments 1 and 2A) were more strongly decreasing in blocks with a high compared with a low proportion of incongruent (inducer) trials. Interestingly, however, there was no evidence for slope differences as a function of the PC manipulation with auditory distractors (Experiments 1 and 2B). Differences in delta plot slopes have been often used to infer differences in conflict and/or distractor processing, such as the type of conflict (e.g., Wiegand & Wascher, 2005) and/or the timing of cognitive control (e.g., distractor suppression, cf. Ridderinkhof et al., 2004). The distinct delta plot slope patterns observed in the present study thus provide another marker for distractor modality-specific global behavioral adaptation effects and theoretical accounts of the proportion congruent effect need to explain why the behavioral effects fluctuate differently over time with different distractor modalities. For example, assuming that the slope of delta plots reflects the time-course of distractor activation (e.g., Ellinghaus & Miller, 2018; Mittelstädt et al., 2022a, b, c; Ridderinkhof et al., 2004; Ulrich et al., 2015), it is possible that a PC modulation in the presence of visual distractors affects not only the strength but also the speed of distractor suppression (cf. Mittelstädt et al., 2022c). Another possibility to consider, which is not mutually exclusive, is that the different slopes observed in delta plots can be attributed to the relative speed of target-to-distractor processing (Heuer et al., 2023; Mackenzie et al., 2022). For example, according to the staggered-onset account, negative slopes occur when distractor processing precedes target processing, while positive slopes occur when distractor processing is slower or comparable in speed to target processing (Heuer et al., 2023). In the case of visual distractors, which share the same modality and are more beneficial in the high PC condition, this may accelerate distractor processing relative to target processing, resulting in more negative slopes in this condition.

### Exploring local behavioral adaptations: Congruency sequence effects

While the present experiments clearly show that the specific distractor modalities act as a boundary condition of global behavioral adaptation effects (as measured via the PCE), it was unclear whether the distractor modality-specific account extends to local behavioral adaptations (as measured via the CSE). In additional (not preregistered) analyses, we explored the CSE as a function of distractor modalities in each experiment (see Appendix Fig. 8, 9 and 10). In Experiment 1, CSEs were present for both distractor modality repetition (visual-visual and auditory-auditory) and distractor modality switches (visual-auditory and auditory-visual), though smaller in the latter. Thus, Experiment 1 provides evidence for distractor-modality-general local behavioral adaptation effects when the PC manipulation was equally applied to both distractor modalities. While this might imply that at least partially different mechanisms underly local and global behavioral adaptations (e.g., Funes et al., 2010; Luo et al., 2022), further studies are needed to better understand boundary conditions of potential CSE transfer across distractor modalities.

First, there are also some recent studies showing a lack of CSE across trials with different target and/or distractor modalities (e.g., Kreutzfeldt et al., 2016; Li et al., 2021; Yang et al., 2017) and one may speculate that local behavioral effects can only occur across different distractor modalities when the target modality remains constant as in the present study. Second, the pattern becomes more complex in the present study when considering the findings of Experiments 2A (inducer: visual) and 2B (inducer: auditory), in which the PC manipulation was only implemented for the inducer distractor modality. While clear CSE effects could be observed for distractor modality-repetitions in Experiments 2A and 2B independent of inducer and diagnostic distractor modality, distractor modality switches showed some unexpected distractor modality-specific effects. Specifically, for distractor modality-switches in current auditory-based distractor trials in both Experiment 2A and 2B, there were standard CSEs in ERs (but only significant in Experiment 2B). However, there were significantly reversed CSE in RTs (i.e., larger CE after incongruent compared with congruent trials). For distractor modality-switches in visual-based distractor trials, a similar dissociation in RTs and accuracy was present in Experiment 2B, but there was no evidence for any CSE modulation in Experiment 2A. While we do not have a ready theoretical explanations for this pattern, one may speculate that the distractor modality-specific global expectancies due to the PC manipulation may have somehow overshadowed more standard CSE patterns seen under a balanced setting as in Experiment 1. Thus, future studies may more directly investigate the interplay of global and local behavioral adaptations effects and their underlying mechanisms with multimodal information. For example, it remains to be seen whether a different pattern would emerge when controlling for contingency learning (e.g., Braem et al., 2019) or when orthogonally varying both distractor and target modalities.

### Conclusion

Across three experiments, we investigated the effects of PC manipulations in an Eriksen flanker task with visual targets and randomly varying visual or auditory distractors. In all experiments, there were global behavioural adaptation effects (PCE) for distractor modalities with PC manipulations. Critically, there was no evidence that these adaptation effects transfer across modalities when selectively applying the PC manipulation to either the visual or auditory distractor modality. Thus, these results suggest that distractor modality constrains global behavioral adaptations effects due to for example, the learning of modality-specific memory processes (e.g., distractor–target associations) and/or suppression of modality-specific distractor-based activation.

## Figures and Tables

**Fig. 8:**
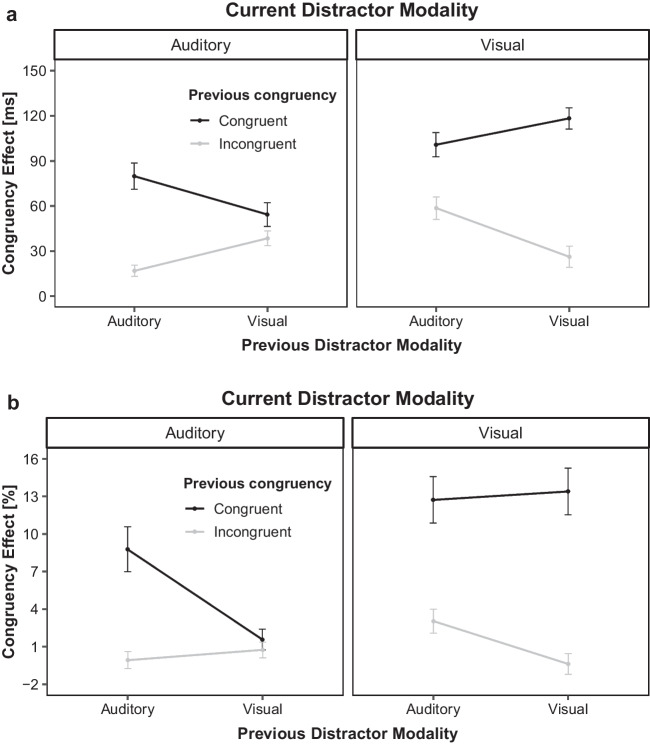
Congruency sequence effects in Experiment 1. Mean congruency effects in reaction time (**a**) and mean error rate (**b**) as a function of congruency in the previous trial (congruent *n* − 1, incongruent *n* − 1) and distractor modality in the previous trial (auditory *n* − 1, visual *n* −1) separately for whether the current distractor modality was auditory (left) or visual (right). Error bars indicate 1 standard error (*SE*) of the corresponding means

**Fig. 9:**
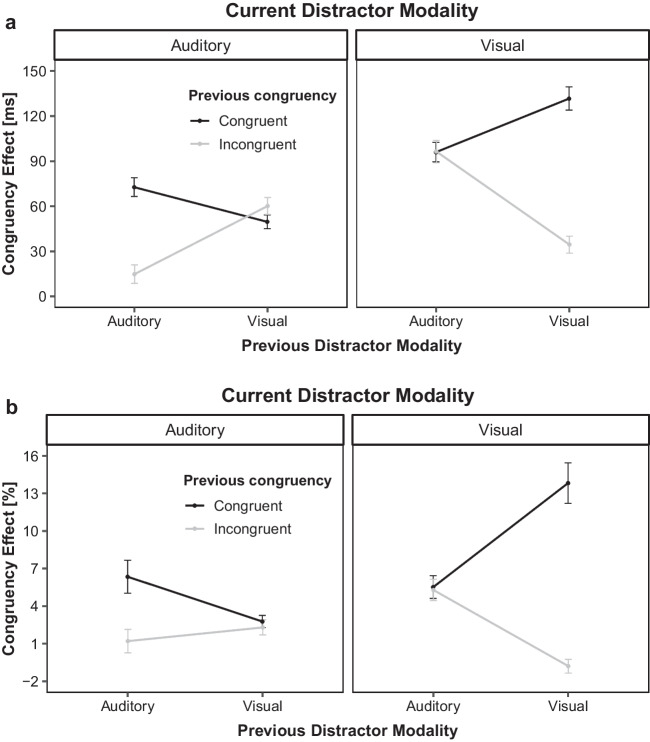
Congruency sequence effects in Experiment 2A. Mean congruency effects in reaction time (**a**) and mean percentage error (**b**) as a function of congruency in the previous trial (congruent *n* − 1, incongruent *n* − 1) and distractor modality in the previous trial (auditory *n* − 1, visual *n* − 1) separately for whether the current distractor modality was auditory (left panel) or visual (right panel). Inducer modality was visual. Error bars indicate 1 standard error (*SE*) of the corresponding means

**Fig. 10:**
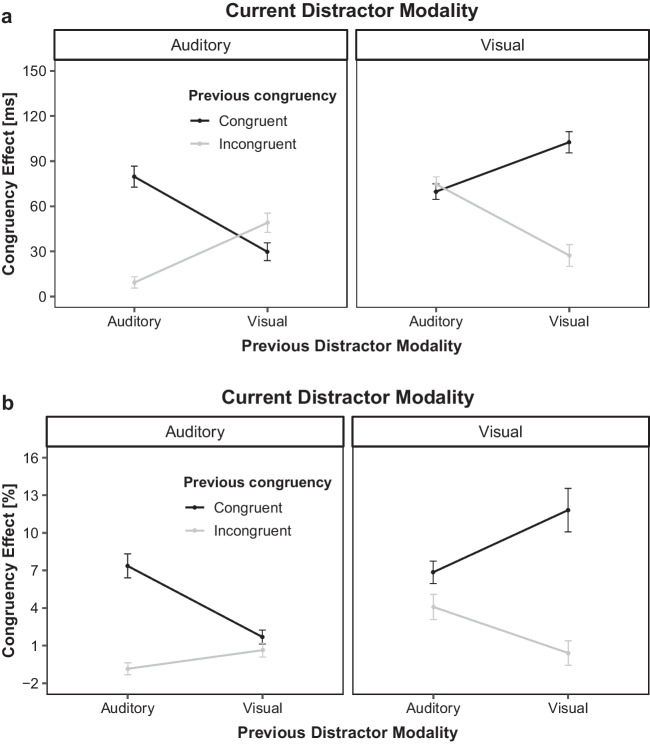
Congruency sequence effects in Experiment 2B. Mean congruency effects in reaction time (**a**) and mean percentage error (**b**) as a function of congruency in the previous trial (congruent *n* − 1, incongruent *n* − 1) and distractor modality in the previous trial (auditory *n* − 1, visual *n* − 1) separately for whether the current distractor modality was auditory (left panel) or visual (right panel). Inducer modality was auditory. Error bars indicate 1 standard error (*SE*) of the corresponding means

## Appendix 1

### Mean reaction time and error rates

In this appendix, we present complementary analyses on mean reaction time (RT) and error rates (ER)

**Table 3:** Mean reaction time (RT, in ms) and percentage of error rates (ER) in congruent and incongruent trials, as well as congruency effects for RT (ΔRT) and ER (ΔER), for each (Distractor) Modality and Proportion of Congruency (PC) in Experiment 1.

(Distractor) Modality	PC	RT [ms]			ER [%]		
		Congruent	Incongruent	ΔRT	Congruent	Incongruent	ΔER
Visual	high	374 (64.3)	510 (70.9)	136	2.52 (2.70)	22.43 (18.0)	19.91
	low	439 (59.9)	459 (61.0)	20	7.05 (7.40)	6.35 (4.86)	−0.70
Auditory	high	434 (63.2)	509 (94.1)	75	2.27 (2.36)	9.46 (12.3)	7.19
	low	453 (57.4)	484 (60.8)	32	4.44 (4.90)	4.44 (3.33)	0.00

Standard deviations are given in parentheses. Small deviations from the Δ values reported in the main text are due to rounding error

**Table 4:** Results of two repeated-measures analyses of variance on reaction time (RT) and error rates (ER), with factors: (Distractor) Modality (M), Proportion Congruency (PC), and Congruency (C) in Experiment 1

	RT			ER		
Effect	*F*	*p*	η_p_^2^	*F*	*p*	η_p_^2^
(Distractor) Modality (M)	42.85	<.001	0.49	64.46	<.001	0.59
Proportion congruency (PC)	0.12	.730	0.00	13.65	<.001	0.24
Congruency (C)	132.07	<.001	0.75	35.37	<.001	0.45
M × PC	4.21	.046	0.09	18.39	<.001	0.29
M × C	11.97	.001	0.21	17.00	<.001	0.28
PC × C	238.09	<.001	0.84	50.19	<.001	0.53
M × PC × C	46.57	<.001	0.51	46.85	<.001	0.52

For all effects, dfn = 1 and dfd = 44

**Table 5:** Mean reaction time (RT, in ms) and percentage of error rates (ER) in congruent and incongruent trials, as well as congruency effects for RT (ΔRT) and ER (ΔER), for each (Distractor) Modality and Proportion of Congruency (PC) in Experiment 2A with visual inducer

(Distractor) Modality	PC	RT [ms]			ER [%]		
		Congruent	Incongruent	ΔRT	Congruent	Incongruent	ΔER
Visual	high	372 (59.7)	520 (87.2)	148	1.43 (1.29)	22.5 (19.6)	21.1
	low	468 (65.1)	475 (76.3)	8	6.71 (7.00)	4.50 (3.63)	−2.21
Auditory	high	472 (72.6)	524 (84.9)	52	1.22 (1.81)	4.31 (4.94)	3.09
	low	496 (78.9)	550 (87.8)	54	2.12 (2.54)	4.79 (4.39)	2.67

Standard deviations are given in parentheses. Small deviations from the Δ values reported in the main text are due to rounding error

**Table 6:** Results of two repeated-measures analyses of variance on reaction time (RT) and error rates (ER), with factors: (Distractor) Modality (M), Proportion Congruency (PC), and Congruency (C) in Experiment 2A with visual inducer.

	RT			ER		
Effect	*F*	*p*	η_p_^2^	*F*	*p*	η_p_^2^
(Distractor) Modality (M)	182.64	<.001	0.78	61.71	<.001	0.55
Proportion Congruency PC)	37.65	<.001	0.42	21.26	<.001	0.29
Congruency (C)	218.75	<.001	0.81	62.29	<.001	0.55
M × PC	0.09	.765	0.00	39.60	<.001	0.44
M × C	18.26	<.001	0.26	24.78	<.001	0.33
BPC × C	166.13	<.001	0.77	64.77	<.001	0.56
M × PC × C	290.28	<.001	62.42	46.85	<.001	0.55

For all effects, dfn = 1 and dfd = 51

**Table 7:** Mean reaction time (RT, in ms) and percentage of Error rates (ER) in congruent and incongruent trials, as well as congruency effects for RT (ΔRT) and ER (ΔER), for each (Distractor) Modality and Proportion of Congruency (PC) in Experiment 2B with auditory inducer

(Distractor) Modality	PC	RT [ms]			ER [%]		
		Congruent	Incongruent	ΔRT	Congruent	Incongruent	ΔER
Visual	high	412 (68.7)	482 (81.0)	70	3.94 (4.00)	9.91 (6.32)	5.96
	low	416 (64.5)	486 (72.6)	70	5.05 (5.12)	10.2 (8.23)	5.03
Auditory	high	416 (73.8)	500 (103)	84	2.27 (2.29)	10.3 (8.40)	8.02
	low	441 (72.0)	457 (64.7)	16	3.55 (4.55)	3.27 (2.71)	-0.29

Standard deviations are given in parentheses. Small deviations from the Δ values reported in the main text are due to rounding error

**Table 8:** Results of two repeated-measures analyses of variance on reaction time (RT) and error rates (ER), with factors: (Distractor) Modality (M), Proportion Congruency (PC), and Congruency (C) in Experiment 2B with auditory inducer

	RT			ER		
Effect	*F*	*p*	η_p_^2^	*F*	*p*	η_p_^2^
(Distractor) Modality (M)	1.22	.274	0.02	25.64	<.001	0.31
Proportion Congruency (PC)	0.30	.583	0.01	9.53	.003	0.14
Congruence (C)	270.27	<.001	0.83	82.70	<.001	0.59
M × PC	9.96	.003	0.15	20.50	<.001	0.26
M × C	10.65	.002	0.16	3.20	.079	0.05
BPC × C	46.47	<.001	0.45	51.21	<.001	0.47
M × PC × C	58.85	<.001	0.51	19.31	<.001	0.25

For all effects, dfn = 1 and dfd = 57

## Appendix 2

### Additional analyses regarding local processing adjustments in Experiment 1, 2A, and 2B: Congruency sequence effects (CSE)

In this appendix, we provide the results of additional analyses exploring the congruency sequence effect (CSE) as a function of distractor modality and PC manipulation. For this analysis, we employed the same data preparation procedure as described in the main text. Note that qualitatively similar results were obtained when post-error trials were additionally excluded. Because there was a significant three-way interaction in all experiments between the factors current distractor modality, previous congruency and previous distractor modality, we conducted separate ANOVAs for each current distractor modality. Figures 8, 9 and 10 display the corresponding mean congruency effects for each experiment.

### Experiment 1

First, we investigated potential RT-based sequential congruency effects (Fig. 8a). For the ANOVA with current visual distractors (right panel), there was a significant main effect of previous congruency, with larger congruency effects when the previous trial was congruent compared with incongruent (110 ms versus 42 ms), *F*(1, 44) = 230.03, *p* < .001, η_p_^2^ = 0.84, which was further modulated by previous distractor modality, *F*(1, 44) = 45.25, *p* < .001, η_p_^2^ = 0.51. Again, the CSE was larger when preceded by the same compared with the different distractor modality (118 ms − 26 ms = 92 ms versus 101 ms − 59 ms = 42 ms), but significant for both previous distractor modality trial types (*p*s < .001).

For the ANOVA with current auditory distractors (left panel), there was also a significant main effect of previous congruency, reflecting larger congruency effects when the previous trial was congruent compared with incongruent (67 ms versus 28 ms), *F*(1, 44) = 51.00, *p* < .001, η_p_^2^ = 0.54. Furthermore, the interaction between previous distractor modality and previous congruency was significant, *F*(1, 44) = 28.81, *p* < .001, η_p_^2^ = 0.40. The CSE was larger when the previous distractors were auditory (modality repetition) compared with visual (modality switches) (80 ms – 17 ms = 63 ms versus 54 ms − 39 ms = 15 ms). Pairwise comparison revealed that the CSEs were significant for both trials preceded by auditory (*p* < .001) and visual distractors (*p* = .017), though smaller in the latter one.

Now we look at ER-based sequential congruency effects (Fig. 8b). For visual distractor trials (right panel), there were significant main effects of previous congruency (13.06% versus 1.33% for previous congruent and incongruent, respectively), *F*(1, 44) = 65.22, *p* < .001, η_p_^2^ = 0.60, and previous distractor modality (previous auditory: 7.88%; previous visual: 6.51%), *F*(1, 44) = 4.49, *p* = .040, η_p_^2^ = 0.09. The interaction was again significant, *F*(1, 44) = 9.31, *p* = .004, η_p_^2^ = 0.17. Pairwise comparison revealed that the CSE were significant for both trials preceded by auditory and visual distractors (*p*s < .001). For auditory distractors (left panel), the previous congruency had a main effect on ER-based congruency effects, indicating larger congruency effects when the previous trial was congruent compared with incongruent (5.17% versus 0.34%), *F*(1, 44) = 13.51, *p* = .001, η_p_^2^ = 0.23. There was also a significant main effect of previous distractor modality, reflecting larger congruency effects when the previous distractor modality was auditory than visual (4.35% versus 1.16%), *F*(1, 44) = 17.60, *p* < .001, η_p_^2^ = 0.29. The significant interaction indicated a larger CSE for distractor modality repetition (*p* < .001) than distractor modality switches (*p* = .343), *F*(1, 44) = 16.70, *p* < .001, η_p_^2^ = 0.28.

### Experiment 2A: Visual inducer

First, we look at RT-based sequential congruency effects (Fig. 9a). For the ANOVA with current auditory distractor modality (left panel), there were significant main effects of previous congruency (previous congruent: 61 ms; previous incongruent; 37 ms), *F*(1, 51) = 31.01, *p* < .001, η_p_^2^ = 0.38, previous distractor modality (previous visual: 55 ms; previous auditory; 44 ms), *F*(1, 51) = 4.15, *p* = .047, η_p_^2^ = 0.08, as well as a significant interaction, *F*(1, 51) = 59.26, *p* < .001, η_p_^2^ = 0.54. The CSE was only present when the previous distractor modality was the same as the current one auditory (diagnostic) (73 ms − 15 ms = 58 ms; *p* < .001), but not (and even reversed for distractor modality switches (50 ms − 60 ms = −10 ms, *p* = .040). For the ANOVA with current visual-inducer distractors (right panel), there were significant main effects of previous congruency (previous congruent: 114 ms; previous incongruent; 65 ms), *F*(1, 51) = 323.30, *p* < .001, η_p_^2^ = 0.86, and previous distractor modality (previous visual: 83 ms; previous auditory; 96 ms), *F*(1, 51) = 14.20, *p* < .001, η_p_^2^ = 0.22. The interaction between previous distractor modality and previous congruency was also significant, *F*(1, 51) = 168.21, *p* < .001, η_p_^2^ = 0.77. Critically, in contrast to Experiment 1, the CSE was only significantly different when preceded by the same (visual) distractor modality (131 ms versus 34 ms; *p* < .001), but not when preceded by the different (auditory) distractor modality (both 96 ms; *p* = .910).

Now we look at ER-based sequential congruency effects (Fig. 9b). For current auditory-diagnostic distractors (left panel), there was a significant main effect of previous congruency (4.6% versus 1.8%), *F*(1, 51) = 10.16, *p* = .002, η_p_^2^ = 0.17. Previous distractor modality had no effect on ER-based CSEs, *F*(1, 51) = 2.38, *p* = .0129, η_p_^2^ = 0.04. As before, the interaction between previous distractor modality and previous congruency was also present in trials with current auditory distractors, *F*(1, 51) = 7.03.46, *p* = .011, η_p_^2^ = 0.12. Pairwise comparisons revealed that the CSE’s were only significant when auditory trials were preceded by the same auditory distractor modality (6.4% versus 1.2%; *p* = .003), but not when preceded by visual ones (2.8% versus 2.3%; *p* = .47). Similar to auditory distractor trials, there was a main effect of previous congruency visible in the congruency effects when current distractors were visual (right panel), *F*(1, 51) = 63.80, *p* < .001, η_p_^2^ = 0.56. This CSE was larger when the previous trial was congruent compared with incongruent (9.67% versus 2.25%). Similar to current auditory trials, previous distractor modality did not affect the CSE in trials with visual flankers when the inducer distractor modality was visual, *F*(1, 51) = 3.34, *p* = .074, η_p_^2^ = 0.06. However, the interaction between previous distractor modality and previous congruency was again significant, *F*(1, 51) = 87.11, *p* < .001, η_p_^2^ = 0.63. Pairwise comparisons revealed that the CSEs were only significant when the distractor modality did not change (13.8% versus −0.8%; *p* < .001), but not when preceded by an auditory distractor (5.5% versus 5.3%; *p* = .75).

### Experiment 2B: Auditory inducer

Again, first looking at RT-based sequential congruency effects for visual inducer (Fig. 10a). For the ANOVA with current auditory distractor modality (left panel) there, as seen in Experiment 1 and 2A, was a significant main effect of previous congruency, reflecting larger congruency effects when the previous trial was congruent compared with incongruent (55 ms versus 29 ms), *F*(1, 57) = 65.55, *p* < .001, η_p_^2^ = 0.53. Furthermore, the interaction between previous distractor modality and previous congruency was significant, *F*(1, 57) = 72.26, *p* < .001, η_p_^2^ = 0.56. Pairwise comparisons revealed that the CSE was significant for both trials preceded by auditory (80 ms versus 9 ms; *p* < .001) and visual distractors (30 ms versus 49 ms; *p* = .001) though reversed in the latter one. This means that there was a reversed CSE in the auditory inducer experiment when the previous distractor modality was visual (diagnostic) and the current one was auditory (inducer), similar to findings with the visual inducer. Regarding the ANOVA with current visual distractors (right panel), there was once again a significant main effect of previous congruency, being bigger when the previous trial was congruent compared with incongruent (86 ms versus 51 ms), *F*(1, 57) = 39.90, *p* < .001, η_p_^2^ = 0.41. Similar to the results in Experiment 2A, there was a significant interaction between previous distractor modality and previous congruency, *F*(1, 57) = 43.16, *p* < .001, η_p_^2^ = 0.43. Interestingly, the CSE was only significantly different when preceded by the same (visual) distractor modality (103 ms versus 27 ms; *p* < .001), but not when preceded by the different (auditory) distractor modality (70 ms versus 75 ms; *p* = .350).

Now we look at ER-based sequential congruency effects (Fig. 10b). For current auditory distractor trials (left panel), there was a main effect of previous congruency, *F*(1, 57) = 57.19, *p* = .001, η_p_^2^ = 0.50, being larger when the previous trial was congruent compared with incongruent (4.5% versus −0.1%). There was a main effect of previous distractor modality, *F*(1, 57) = 19.90, *p* < .001, η_p_^2^ = 0.26, being more pronounced when the previous distractor modality was auditory (inducer) (3.3%) compared with when visual (1.2%). The interaction between previous distractor modality and previous congruency was also present in trials with current auditory distractors, *F*(1, 57) = 22.46, *p* < .001, η_p_^2^ = 0.28. Pairwise comparisons revealed that the CSEs were significant for both trials preceded by the same auditory distractor modality (1.7% versus 0.6%) and trials preceded by visual distractors (7.4% versus −0,8%; both *p*s < .001). Again, this effect was smaller when distractor modality changed.

Similar to auditory distractor trials, there was a main effect of previous congruency visible in the congruency effects with current visual distractors (right panel), *F*(1, 57) = 50.89, *p* < .001, η_p_^2^ = 0.47. This CSE was again larger when the previous trial was congruent compared with incongruent (9.3% versus 2.2%). In contrast to current auditory trials, previous distractor modality did not affect the CSE in trials with visual flanker, *F*(1, 57) = 0.40, *p* = .528, η_p_^2^ = 0.01. However, the interaction between previous distractor modality and previous congruency was again significant, *F*(1, 57) = 16.31, *p* < .001, η_p_^2^ = 0.22. Pairwise comparisons revealed that the CSEs were significant for both visual trials preceded by auditory (6.9% versus 4.1%; *p* = .004) and visual distractors (11.8% versus 0.4%; *p* < .001), though once again smaller when modality changed.

## Footnotes

1 To be precise, we will use the term (global) behavioral adjustments to describe a change in behavior visible in error rates and reaction time that is based on the implemented global (proportion of congruency) manipulation.

2 It should be also emphasized that in the present study as well as in other studies both accounts could explain to the observed PCEs (e.g., Bausenhart et al., 2021; Shichel & Goldfarb, 2022). Note also that the term *global behavioral adaptations* (or *adjustments*) refers to behavioral changes to environments with different congruency proportions and hence does not necessarily imply that, for instance, the underlying mechanisms in producing these behavioral adjustments operate in a global manner.

3 As described in the Method sections of the present web-based experiments, participants were required to respond to the distractor modality in 5% of the trials. This procedural aspect was implemented to ensure that participants kept their sound system on. Moreover, we also reasoned that this would generally produce larger congruency effects, which should help to detect potential significant modulations. However, one might also argue that this leads to the formation of modality-based task-sets and therefore limits the potential transfer of the PCE across modalities if the task-set account of the CSE (Grant et al., 2020) also applies to the PCE.

4 For example, consider that the PCE may reflect processes associated to the CSE because in blocks with a high (low) proportion of congruent trials, trials are more likely preceded by congruent (incongruent) trials

5 Note that, in these experiments, the overall proportion of congruent trials for high versus low PC blocks was 75% versus 25% due to our use of fewer diagnostic trials and catch trials (see Table 2).

6 For example, detecting a PCE with visual distractors of similar effect size (η_p_^2^ = .95) as Jost et al. (2022) with a power of 95% would have suggested that four participants are already sufficient.

7 Of course, online experiments, including the ones of the present study, do not allow for the same level of control over stimulus presentation timing of stimuli, and apparatus (e.g., which monitor or headphones/speakers were used) as laboratory-based experiments. However, we do not see any reason that this affects or limits the conclusions drawn from our study. Consider also that research has shown that internet-based mental chronometry studies produce performance measures comparable to those collected in the laboratory (e.g., Miller et al., 2018). Moreover, any potential (presumably primarily unsystematic) additional noise in an online setting should have affected all conditions equally, given that they were tested within subject. Finally, the substantial mean congruency effects observed in both modalities suggest that both types of distractors significantly affected behavior and the additional distribution analyses even revealed that these effects were present throughout the RT distribution (e.g., for fast and slow responses, see also the General Discussion).

8 Results in all experiments were very similar when using more conservative criteria (e.g., 40% or 30%).

9 We decided to present the data using mean congruency RT and ER effects as the dependent variable to make it more accessible for readers. However, it is worth noting that similar conclusions can also be drawn based on mean RT and ER. Furthermore, the delta plots provide evidence that the observed results are not dependent on general speed differences in conditions with auditory or visual distractors (see also section “Delta plots and their implications” in the General Discussion).

10 As mentioned earlier, this is simply a description of the results without implying any specific mechanisms. The behavioral adaptation effect refers to a change in behavior that is visible in error rates and reaction time and is based on the proportion of congruent trials (or to the sequence of congruency, see below).

11 To explore whether higher-level cognitive control was involved in the observed adaptations, we examined the potential presence of an asymmetrical list shifting effect (as described in Abrahamse et al., 2013). According to cognitive control accounts, shifting from high-PC to low-PC blocks should result in a greater decrease in congruency effects than shifting from low-PC to high-PC blocks and the presence of such asymmetrical shifts cannot be solely explained by contingency learning. Consistent with cognitive control accounts, the PCE for auditory (inducer) distractor trials was significantly modulated in this manner in Exps. 1 and 2b. Interestingly, we did not observe this interaction for visual distractor trials in Experiment 1 and 2a, suggesting that the involvement of cognitive control and contingency learning may differ to some extent depending on the distractor modality.

12 It is worth noting that these visual inspections are consistent with the corresponding inferential statistics. Specifically, exploratory analyses using paired *t* tests indicated no significant differences in the congruency effects between high and low PC for diagnostic trials when examined separately at each percentile. However, for inducer trials, the differences were significant at each percentile.
